# Optimized cesarean techniques, IVF use, and foster strain selection enhance germ-free mouse production efficiency

**DOI:** 10.1038/s41598-025-05411-4

**Published:** 2025-07-26

**Authors:** Yuting Yang, Weimin He, Jingqiang Li, Jinhui Wang, Yuyan Tang, Haitao Shang

**Affiliations:** 1 Germ-Free Mouse Research Facility, The First Affiliated Hospital of Sun Yat-sen University, Guangzhou, China; 2 Shanghai Tenth People’s Hospital, Tenth People’s Hospital of Tongji University, Shanghai, China

**Keywords:** Microbiology, Animal breeding

## Abstract

This study aims to refine sterile cesarean section techniques, in vitro fertilization (IVF) and foster mother selection strategies to enhance the efficiency of germ-free (GF) mouse production. We conducted the following analyses: (1) comparing different cesarean section methods, (2) evaluating the impact of natural mating versus in vitro fertilization (IVF) for obtaining donor mice, and (3) assessing maternal care among three inbred strains (C57BL/6J, BALB/c, NSG) and one outbred strain (KM) as GF foster mothers. The results showed that optimizing surgical methods which preserve the female reproductive tract during cesarean section (FRT-CS), significantly improved fetal survival rates while maintaining sterility. IVF enabled precise control over donor delivery dates, enhancing experimental reproducibility. Among GF foster strains, BALB/c and NSG mice exhibited superior nursing and weaning success, whereas C57BL/6J had the lowest weaning rate, in stark contrast to findings on maternal care in SPF C57BL/6J foster mothers. These findings offer practical guidance for optimizing GF mouse production, improving the efficiency of obtaining germ-free mice through cesarean derivation.

**Supplementary Information:**

The online version contains supplementary material available at 10.1038/s41598-025-05411-4.

## Introduction

In 1885, Louis Pasteur initially proposed the concept of GF animals, although the research results represented that bacteria-free life was impossible. A decade later, the first GF mammal (a guinea pig) was generated at Berlin University but survived only 13 days due to inadequate sterile techniques and nutritional support^1^. The breakthrough came when Gustafsson successfully obtained GF rats via sterile C-section, followed by Pleasants producing GF mice in 1959. Advancements in isolator technology and artificial rearing methods subsequently enabled the survival and breeding of GF animals, including mice, rats, rabbits, pigs, and goats^2^. These innovations paved the way for maintaining GF colonies for microbiome research.

Germ-free mice and genetically engineered GF mice are the irreplaceable animal model to study the interaction of microbiome and human genes on human health and disease. Improving the efficiency of generating germ-free mice can significantly shorten the time required to produce germ-free mice. Additionally, it enables the rapid recovery of germ-free colonies in the event of contamination in germ-free mouse production facilities. Aseptic embryo transfer and sterile cesarean section are the two primary methods for generating GF mice. An aseptic embryo transfer must be conducted entirely within a sterile isolator, necessitating the integration of a stereomicroscope^3^. In this process, GF females are mated with pre-vasectomized GF males to obtain pseudopregnant recipients, while donor embryos are derived from specific pathogen-free (SPF) mice through in vitro fertilization (IVF)^4^. The embryos are then transferred into the recipient via surgical placement into the infundibulum or uterus, or through cervical injection^5^. Additionally, embryo survival rates are relatively low due to surgical constraints, with only approximately 50% of transferred embryos resulting in live births^6^.

The “sterile womb hypothesis” posits that the placental epithelium serves as a barrier protecting the fetus from microbial exposure, supporting the consensus that term fetuses develop in a sterile intrauterine environment^7^. The caesarean section rederivation is based on this theory and still be considered as the golden method of obtaining germ-free mice. The fetuses are delivered by sterile C-section from SPF donor female mice, and the uterine sac is removed from donor mice to disinfectant^8^. Then immediately transfer into a sterile polyvinyl chloride isolator and peel off the fetus. The amniotic membrane was then incised with surgical scissors to expose the pup, followed by umbilical cord cutting. Sterile cotton swab was used to wipe up amniotic fluid until spontaneous breathing was noted^9^. Consequently, fetuses are introduced to GF recipient foster mother until maturity^10^. The challenges of aseptic C-section rederivation include variability in donor mice mating times, difficulty in precisely predicting delivery dates, and uncertainty in ensuring adequate maternal care, all of which contribute to inconsistencies in the number of successfully obtained GF pups. To address these issues, our study implemented several improvements. First, we optimized cesarean section techniques to assess their impact on neonatal survival. Second, we integrated the advantages of aseptic embryo transfer while addressing its limitations. We designed an approach using IVF-derived recipient mice to achieve more precise control over delivery timing, followed by C-section to obtain GF pups. Additionally, by systematically recording natural mating and actual delivery times, we aimed to identify patterns in natural birth timing to improve its predictability. Lastly, we evaluated different GF foster strains to identify the most suitable strain for improving post-surgical pup survival rates.

BALB/c and C57BL/6J are the most used inbred strains of laboratory mice. Consequently, previous research on maternal care in SPF inbred mice has primarily focused on these two strains. Findings indicate that C57BL/6J mothers exhibit more active maternal behaviors compared to BALB/c mothers, whereas the milk produced by BALB/c mothers contributes more significantly to pup weight gain^11^. Additionally, no significant differences have been observed in the level of maternal care provided by SPF foster mothers, as they exhibit similar maternal behaviors toward both their biological and cross-fostered pups^12^. Considering the natural variations among different strains, this study includes three inbred strains (C57BL/6J, BALB/c, and NSG) and one outbred strain (KM) as GF foster mothers to evaluate their nursing capabilities.

The production and maintenance of GF mice are costly and require professional technical assistance. The previous protocols for the establishment of the GF mice model mainly focused on equipment improvement and building the living supplies (water, food, bedding) delivering system^13^. This study aims to enhance the efficiency and reproducibility of germ-free (GF) mouse production by optimizing sterile cesarean section techniques, improving donor selection strategies, and identifying the most suitable GF foster strains. Specifically, we refine surgical methods to improve neonatal survival, integrate the advantages of in vitro fertilization (IVF) for precise control over delivery timing, and evaluate the maternal care capabilities of different GF inbred and outbred foster strains. These advancements are expected to minimize variability, reduce pup loss, and improve efficiency in obtaining germ-free mice.

## Method

### Subject

Two original germ-free strains BALB/cAnSlac (BC) and Kunming (KM) were bred in Shanghai Tenth People’s Hospital, Tenth People’s Hospital of TongJi University in Shanghai, China. The SPF NOD/SCID Il2rg^–/–^ (NSG) strain was purchased from the Jackson Laboratory (Bar Harbor, American) and completed biological decontamination by cesarean section. The SPF CD-1 strain was purchased from Genepax Biotechnology Co., Ltd (Guangzhou, China) and used as the recipient mice for embryo transfer. The SPF BALB/c and C57BL/6Slac (C57) were purchased from Shanghai SLAC Laboratory Animal Co., Ltd as donor mouse. The SPF mice were confirmed to be free from pathogenic bacteria virus, and parasitic pathogens listed in supplementary material Table 2.

The house of GF and SPF donor mice and the C-section procedure were completed in a germ-free animal facility at the First Affiliated Hospital, Sun Yat-sen University in Guangzhou, China. All mice were housed with sterile aspen wood shavings (autoclaved before use) as bedding, which was changed once per week. For mating, one male was paired with two female mice per cage. Pregnant donor females were housed individually from the late gestation period through delivery to facilitate accurate monitoring. All mice were maintained under controlled environmental conditions, including a 12-hour light/dark cycle (lights on at 08:00), a constant temperature of 22 ± 2 °C, and a relative humidity of 55%. All animals were given unrestricted access to food (Labdiet 5CJL) and water. All experimental protocols were conducted in compliance with institutional guidelines and were approved by the Animal Care and Use Committee of Shanghai Tenth People’s Hospital (Approval No. SHDSYY-2023-A667). Results are reported in accordance with ARRIVE guidelines.

### Mouse model

#### Comparison of cesarean section techniques

In the first model, we compare two surgical techniques for cesarean section: traditional C-sction (T-CS) and female reproductive tract preserved C-section (FRT-CS). In T-CS, clamps are placed at both the cervix base and the top of the uterine horn, whereas FRT-CS selectively clamps only the cervix base, preserving the entire reproductive tract, including the ovary, uterine horn, uterine junction, and cervix. A total of 80 pregnant SPF mice (40 C57 and 40 BC) were divided equally between the two groups. All donor females were euthanized via cervical dislocation, and C-section was performed under aseptic conditions. Pups were obtained using T-CS and FRT-CS respectively, disinfected with Clidox-S, and transferred to an isolator. Aside from the surgical methods used for fetal extraction, all other procedures remained consistent as previously described. To ensure sterility and pup viability, the entire procedure was completed within 5 min.

### NM versus IVF donor mice for C-Section

In the second model, we evaluated the impact of natural mating (NM) and in vitro fertilization (IVF) on pup survival and contamination rates following cesarean section (C-section). Thirty C57BL/6J female mice underwent NM with a male from the same strain for 72 h. Successful copulation was confirmed by the presence of a vaginal plug, recorded as gestation day 0.5 (G0.5)^14^. Donor mothers were monitored for natural delivery from G18 onward before undergoing FRT-CS. Concurrently, thirty CD-1 female mice served as IVF-derived embryo transfer recipients using C57BL/6J embryos. The implantation of two-cell stage embryos was designated as embryonic day 0.5 (E0.5). These IVF-derived donor mothers underwent pre-labor FRT-CS on the predicted delivery date.

### Maternal care of different strains of GF foster mother

In the third model, the different strains of GF female foster mothers (BC = 15, KM = 15, NSG = 15, C57 = 15) were selected to identify the influence of strain background and fertility on maternal care. All foster mothers were four months old and had previously given birth once.

### Isolator preparation

All germ-free mice were housed in polyvinyl chloride (PVC) isolators, which were purchased from Suzhou Fengshi Laboratory Animal Equipment Co., Ltd. The assembly of the isolator has been described in the previous research report^15^. Due to the heat insulation of the PVC isolator, the heating pad was required to be opened and heated to 40–45 °C for at least 15 min before the C-section began to prevent hypothermia. Clidox-S is used as the chlorine dioxide disinfectant in this study to sterilize tissue samples and disinfect the living environment, it is applied in a 1:3:1 dilution and activated for 15 min before use. Life supplements like food, water, bedding, and surgical instruments are autoclaved at 121 °C for 1200s in advance.

### Sample collection

Two tubes of fecal samples were directly collected from the donor mother and weaned mice, starting from 2 weeks to 4 weeks after C-section, respectively. For culturing, fecal samples were diluted by phosphate-buffered saline (PBS) and cultured in a Columbia blood agar plate under aerobic and anaerobic conditions for 48 h. The left dilution was heat-fixed on the surface of a slide and stained by established procedure. The existence of bacterial morphology could be observed under microscopy.

### PCR

Multiple germ-free research facilities take polymerase chain reaction (PCR) as routine program to test contamination. Bacterial DNA was extracted from fecal sample using E.Z.N.A. Bacterial DNA Kit. Full-length 16S rDNA gene was amplified using 27 F and 1492R primers.

- Forward prime: 5′-AGAGTTTGATCCTGGCTCAG-3′.
- Reverse prime: 3′-GGTTACCTTGTTACGACTT-5′.

PCR reactions were held for 5 min at 95 °C, followed by 35 cycles of 95 °C for 15 s, 55 °C for 30 s, and 72 °C for 45 s. Subsequently, 15µL of each reaction was electrophoresed on a 1.5% agarose gel prepared in 1X TAE buffer at 100 V for 45 min using a BIO-RAD system.

### Statistical analyses

The survival rate (10 min post-C-section) and actual delivery date between NM and IVF groups were analyzed using Student’s *t* test. Comparisons of different strains, survival rates of fostered pups based on postpartum time, and the timing of natural delivery in donor mice were assessed using one-way analysis of variance (ANOVA). The survival rates of IVF donor mice delivered via pre-delivery C-section versus natural delivery were analyzed using the Mann-Whitney U test. All statistical analyses were performed in GraphPad Prism (V.9.0, GraphPad Software, San Diego, California, USA). Data is shown as mean ± standard deviation. *p* value < 0.05 was considered statistically significant.

## Results

### The change of surgery method increases the survival rate

**Fig. 1:**
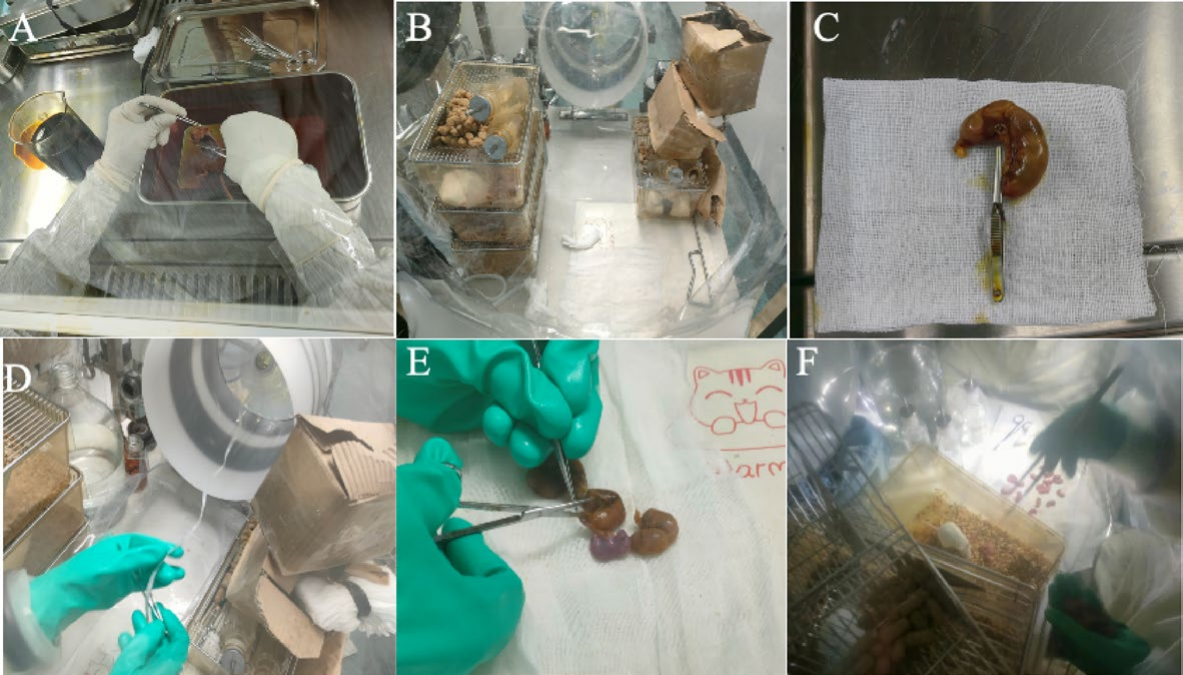
The process of FRT-CS. **A** The abdominal cavity was opened under a sterile laminar flow hood to extract the FRT. **B** The heating pad was settled at 45°C and the space within the isolator was organized. **C** and **D** Clamping only the cervix base, preserving the entire reproductive tract. Transferring FRT into isolator through connected trap with Clidox-S. **E** Open the uterine horns with scissors and clean the pups with sterile cotton swab. **F** The pups were given to a GF foster mother when spontaneous breathing was noted.

**Fig. 2:**
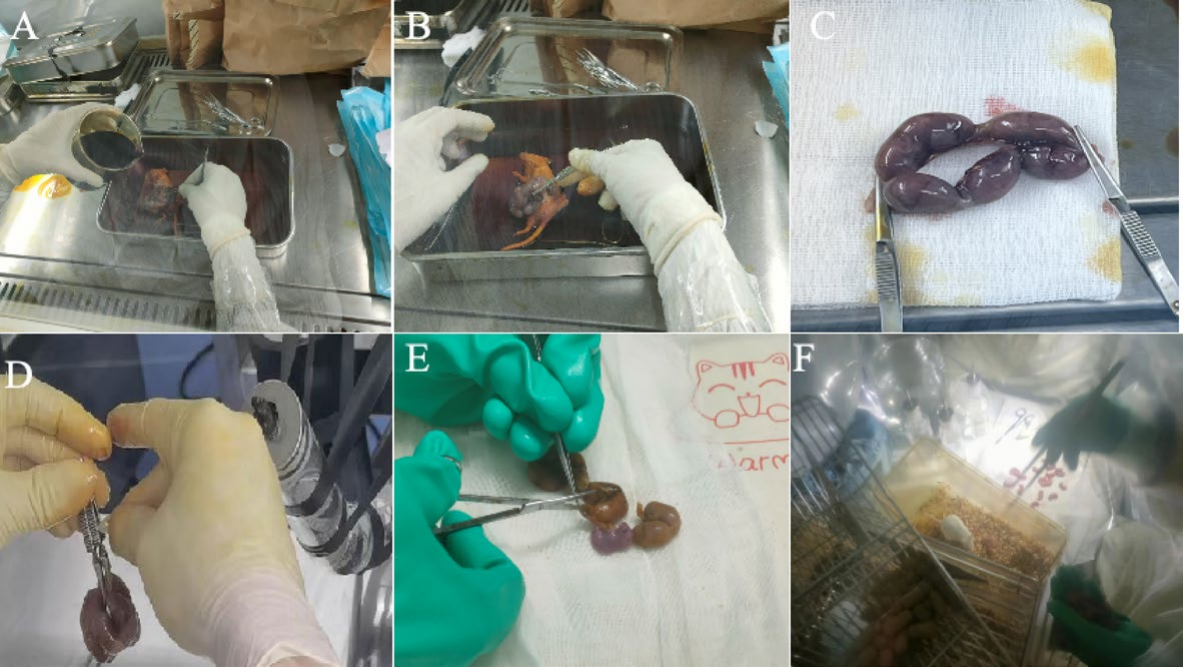
The process of T-CS. **A** The abdominal cavity was opened under a sterile laminar flow hood to extract the FRT. **B** Clamping the base side of the cervix top. **C** Clamping the top of uterine horn. **D** Transferring cervix into isolator through connected trap with Clidox-S. **E** Opening the uterine horns with scissors and cleaning the pups with sterile cotton swab. **F** The pups were given to a GF foster mother when spontaneous breathing was noted.

**Fig. 3:**
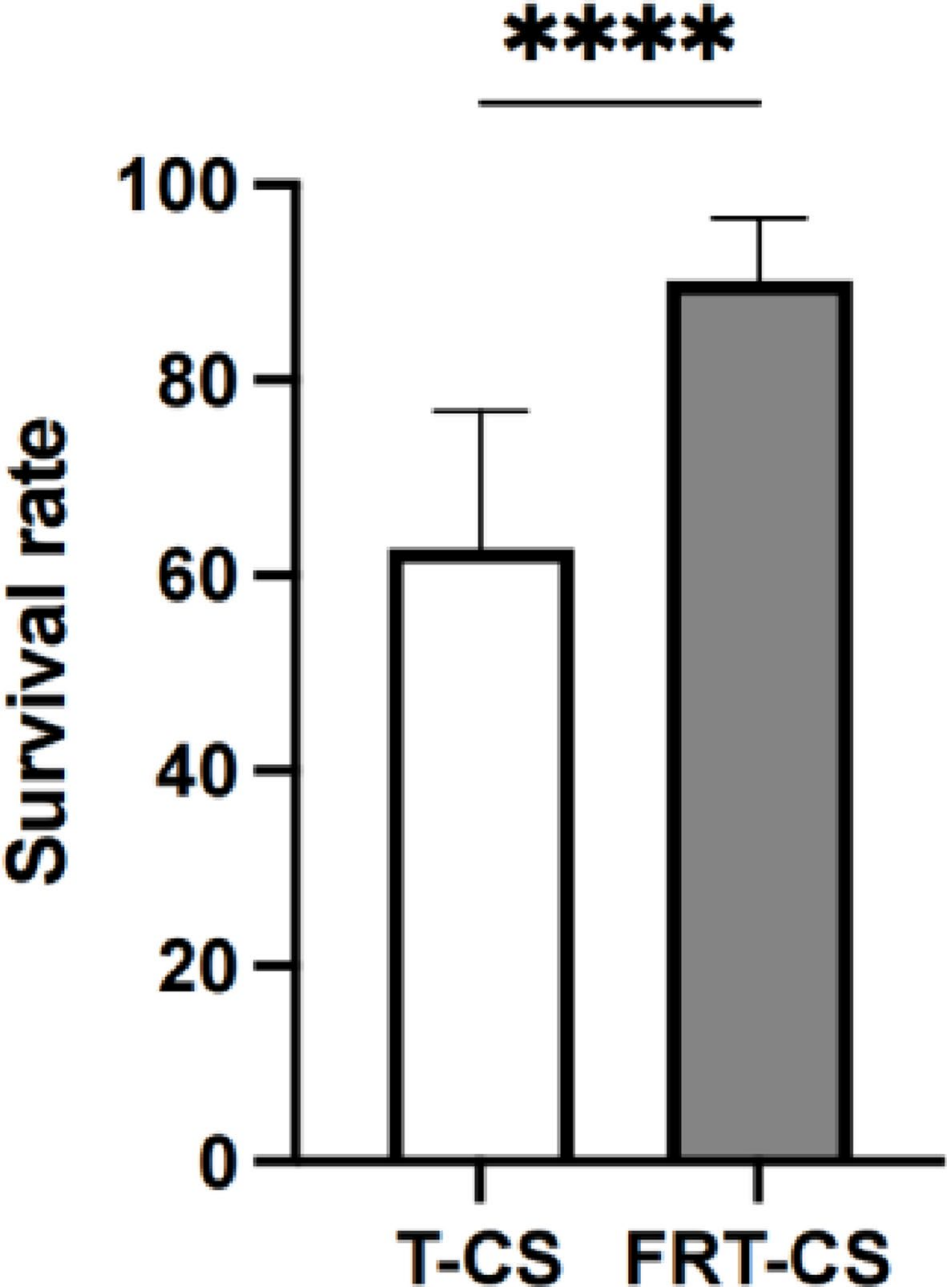
The survival rate (10 min after C-section) and surgery process of sterile caesarean section. The survival rate of two different cesarean section methods, ****p < 0.0001.

To address our hypothesis that the surgery method affects the survival rate, we compared two different anatomical techniques to obtain a uterus. The euthanasia and abdominal cavity opening procedures for both FRT-CS and T-C groups were consistent. Donor female mice were humanely euthanized via cervical dislocation under a laminar flow hood (Figs. 1A, 2A). The entire body surface of the mice was disinfected with povidone-iodine to minimize the risk of contamination. The heating pad was pre-warmed before the dissection began to maintain proper temperature (Fig. 1B). The abdominal cavity was then carefully opened to access the uterus, which was excised and placed on sterile gauze. The uterus was quickly transferred to a sterile connected trap, filling with Clidox disinfectant solution, further transferred to the isolator for processing (Figs. 1D, 2D). Inside the isolator, a surgical scissor was used to carefully incise the uterus, and sterile cotton swabs were used to gently rupture the amniotic membrane (Figs. 1E, 2E). Each pup was then introduced to the GF foster mother until spontaneous breathing was observed (Figs. 1F, 2F). Instead of making incisions on both sides of the uterus (Fig. 2B, C), we opted to clamp only the bottom side of the cervix (Fig. 1C), allowing the uterus to be transferred to the isolator within one minute. Unlike T-CS, which creates three surgical wounds, FRT-CS involves only a single incision, potentially reducing the risk of contamination. Simplifying the surgical procedure reduces the time fetuses remain in the uterus without a blood supply. FRT-CS demonstrated a significantly higher immediate survival rate (*p* < 0.0001) compared to T-CS (Fig. 3).

**Fig. 4:**
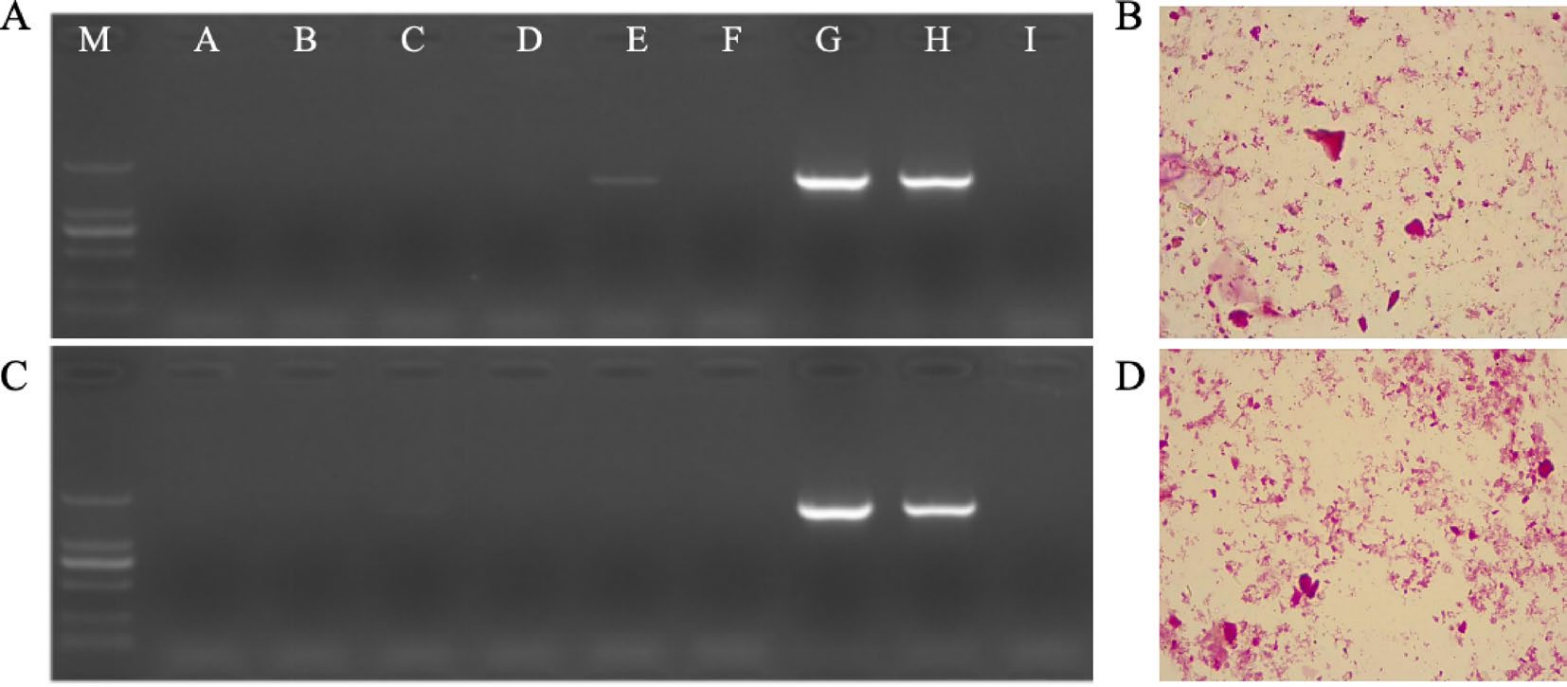
Detection of bacterial contamination after T-CS. **A** 16S rDNA gene PCR of fecal sample from GF foster mother. Lanes A-F: GF mice fecal sample. Lanes G-H: Fecal sample of SPF mice as positive control. Lane I: Nuclease free water. **B** Gram-stained slides of fecal sample from GF foster mother. **C** 16S rDNA gene PCR of fecal sample from weanling pups. **D** Gram-stained slides of fecal sample from GF weanling pups. The complete membrane image, including multiple samples such as the normal detection of germ-free mice, is provided in Supplementary Fig. 2. In the main figure, only the portion relevant to T-CS is shown for clarity.

**Fig. 5:**
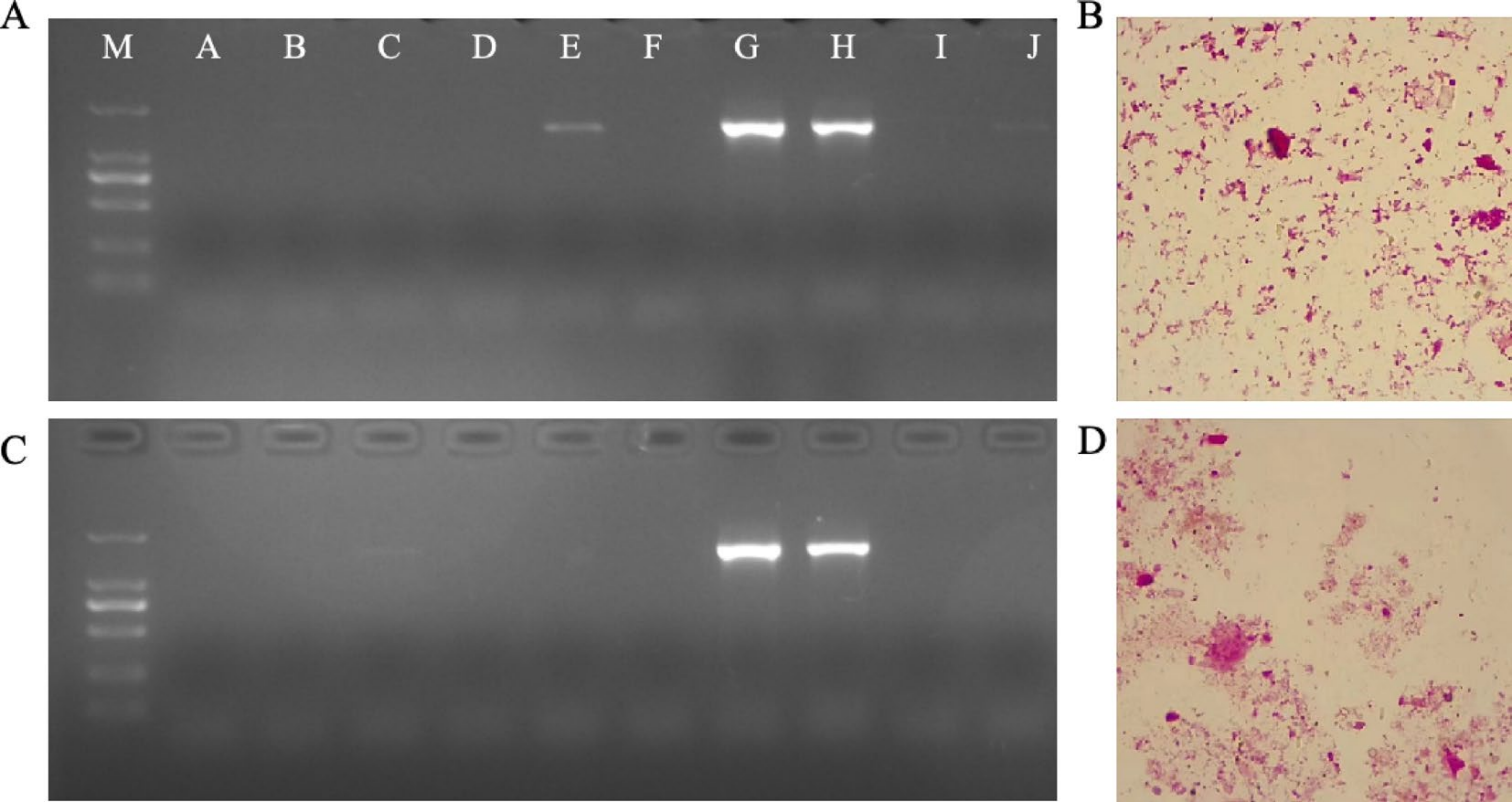
Detection of bacterial contamination after FRT-CS. **A** 16SrDNA gene PCR of fecal sample from GF foster mother. Lanes A-I: GF mice fecal sample. Lane J: Nuclease free water. Lane K: Fecal sample of SPF mice as positive control. **B** Gram-stained slides of fecal sample from GF foster mother. **C** 16S rDNA gene PCR of fecal sample from GF weanling pups. **D** Gram-stained slides of fecal sample from GF weanling pups. The complete membrane image, including multiple samples such as the normal detection of germ-free mice, is provided in Supplementary Fig. 3. In the main figure, only the portion relevant to FRT-CS is shown for clarity.

Contamination monitoring was conducted two weeks post-surgery to avoid triggering pup retrieval behavior in GF foster mothers due to fecal sample collection. The shape and size of bacteria in fecal samples can be distinguished by culturing and staining, the observation of staining is visible under a 1000x oil immersion lens. Gram-stained slides of fecal samples from both GF foster mother (Figs. 4B, 5B) and weanling mice (Figs. 4D, 5D) showed no bacterial morphology. This assessment indicates that the mice are probably in a germ-free state and require further monitoring by PCR. The analysis of gel reveals that the contamination is avoided during the surgery, as no PCR products were detected in GF mouse samples from both T-CS (Fig. 4C) and FRT-CS (Fig. 5C). Low-intensity bands do not necessarily indicate contamination, whereas strong bands were observed in specific pathogen-free (SPF) mice, serving as positive controls (Figs. 4A, 5A). Given the high sensitivity of PCR in detecting conserved regions of 16S rDNA genes, the potential presence of bacterial DNA in food and bedding should be considered^16^.

### IVF as a superior method for obtaining donor mice

**Fig. 6:**
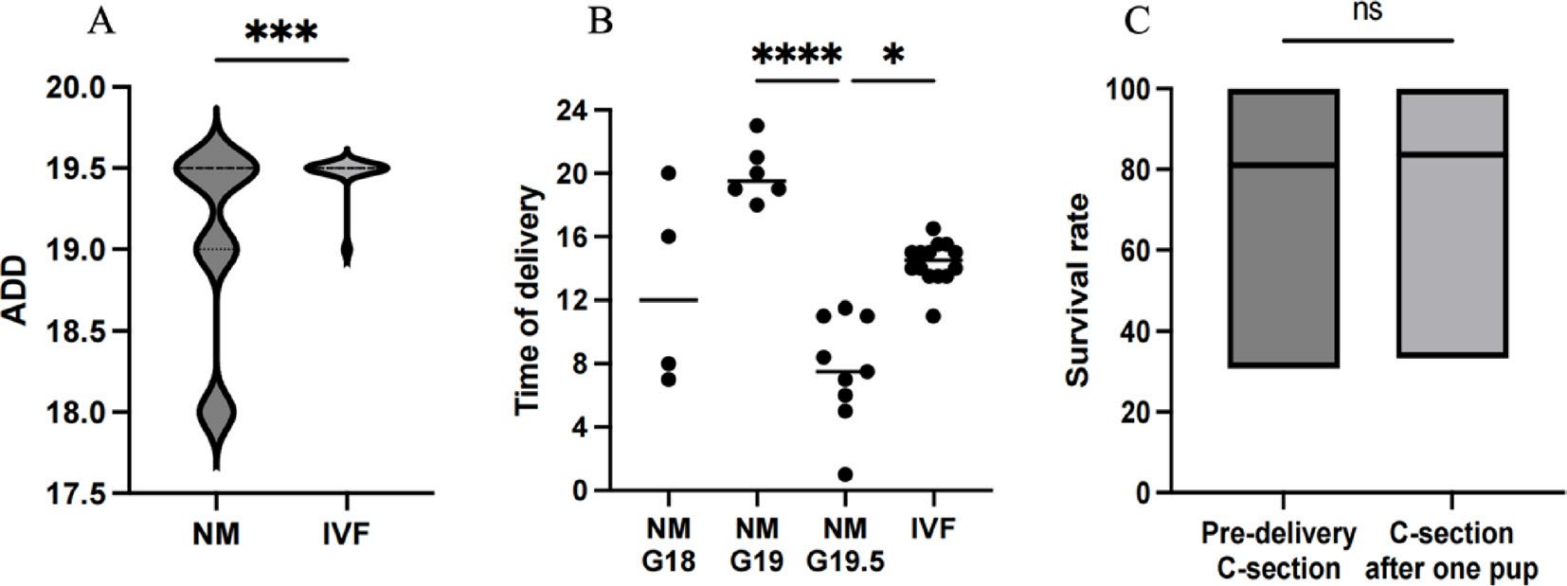
Comparison between natural mating and IVF for obtaining donor mice. **A** Actual delivery date of NM VS IVF, ***p = 0.0004. **B** Timing of natural delivery in donor mice on the EDD, ****p < 0.0001 NM-G19 VS NM-G19.5. **C** Survival rates of IVF donor mice delivered by pre-delivery C-Section versus natural delivery.

The formation of a copulatory plug in female mice is commonly used as an indicator of successful mating. It is typically formed within 24–48 h after mating and naturally dislodges afterward, which makes it hard to observe. In this case, we checked copulatory plugs on the morning of the second day after mating and observed plugs in 76% of the natural mating donor mice (Supplementary Table 1). The average gestation period for mice in natural mating is approximately 19.5 days. The date of vaginal plug detection can be used to estimate the expected delivery date (EDD). Similarly, for mice accepting in vitro fertilization (IVF), the EDD can be estimated based on the timing of embryo transfer. The surrogate mice subjected to IVF embryo transfer exhibited a more concentrated delivery date (*P* < 0.0001), primarily in the afternoon of E19.5 (Fig. 6A). The NM shows a distribution of EDD ranging from G18 to G19.5, with actual delivery times distributed mainly in the night of G19 and the early morning of G19.5 (Fig. 6B). Additionally, performing a C-section before natural delivery has little impact on IVF mice, as there is no significant difference in the survival rates between pups delivered by pre-labor C-section and those delivered naturally and then subjected to C-section (Fig. 6C). Thus, IVF provides greater predictability, allowing precise control over the timing of birth and scheduled pre-delivery C-section once the due date is reached. In contrast, donor mice obtained through natural mating require continuous 24-hour monitoring for spontaneous delivery. Despite estimating the actual delivery date (ADD) based on copulatory plug detection, a significant number of pups remain underdeveloped and fail to survive the surgery.

### The selection of GF foster mother

**Fig. 7:**
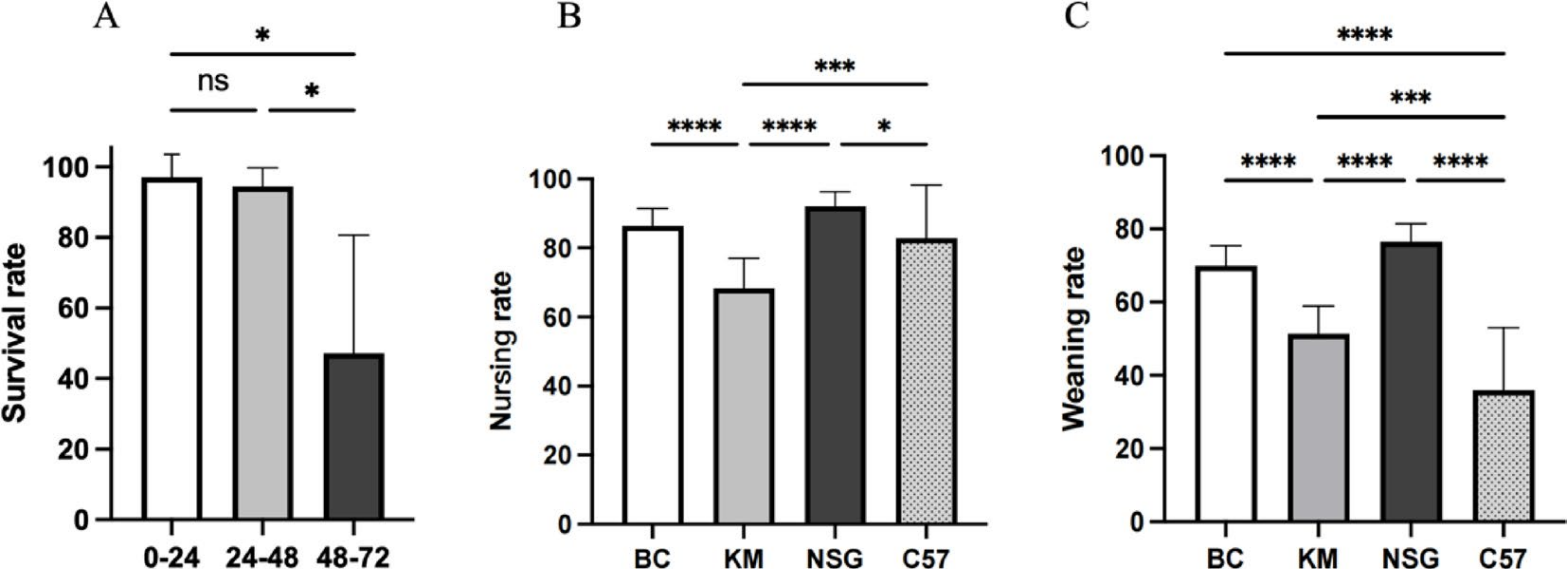
Optimal timing for initiating fostering and the effect of different strain as GF foster mother. **A** Survival rates of fostered pups when nursing begins within 0–72 h postpartum. *p = 0.0140, 0–24 VS 48–72, *p = 0.0364 24–48 VS 48–72. **B** The nursing rate is determined by counting the number of pups that survive 5 days post-C-section across different strains of GF foster mothers. ****p < 0.0001 BC VS KM, ****p < 0.0001 KM VS NSG, ***p = 0.0005 KM VS C57, *p = 0.047 NSG VS C57. **C** The weaning rate is determined by counting the number of pups that survive beyond 3 weeks across different strains of GF foster mothers. ****p < 0.0001 BC VS KM, ****p < 0.0001 BC VS C57, ****p < 0.0001 KM VS NSG, ***p = 0.0002 KM VS C57, ****p < 0.0001 NSG VS C57.

To evaluate the influence of nursing time and strain background on maternal care, we compared four strains of GF mice and the time when nursing begins after natural delivery. We observed that delayed fostering beyond 48 h post-natural delivery significantly reduced fostering success (Fig. 7A). Fostering efficiency varied among strains, with pups showing poor survival under outbred KM foster mothers compared to inbred GF strains. No significant differences were observed in nursing rates among the three inbred GF foster mothers. The number of pups weaned at three weeks was lower than those surviving five days post-C-section, independent of foster mother strain. Notably, C57 exhibited the lowest weaning rate among all strains (Fig. 7B, C).

## Discussion

Our findings demonstrate that modifying the surgical approach significantly improves neonatal survival rates. By adopting the FRT-CS method, which involves a single clamping at the cervix base rather than making incisions on both sides of the uterus, we reduced surgical wounds and shortened the time fetuses remained in the uterus without a blood supply, leading to a significantly higher immediate survival rate compared to the traditional T-CS method (*p* < 0.0001). The sterility of GF pups was confirmed by both Gram staining and PCR analysis, with no detectable bacterial contamination observed post-surgery.

Additionally, our study highlights IVF as a superior method for obtaining donor mice. Unlike natural mating (NM), which presents variability in gestation periods and delivery times, IVF allows for precise control over embryo transfer and expected delivery dates. The concentrated delivery window (*p* < 0.0001) in IVF-derived surrogate mice facilitates pre-labor C-section planning without compromising neonatal survival. Despite recording copulatory plug observations, no consistent pattern could be established for predicting delivery dates in NM mice. Additionally, some NM-derived pups exhibited developmental abnormalities. For certain immunodeficient or genetically engineered mice, issues such as exhibiting challenges with superovulation efficiency or high rates of abnormal oocytes may arise during IVF embryo transfer^17^. In such cases, we recommend using naturally mated method to obtain SPF donor mice.

The selection of GF foster mothers typically prioritizes strains with consistent maternal behavior. KM mice, an outbred strain derived from Swiss mice, are known for larger litter sizes and higher milk production compared to inbred strains^18^. While previous research suggests that maternal behavior varies among different strains, offspring mortality does not differ between inbred and outbred SPF foster mothers^19^. In converse, our results indicate that GF KM mice perform significantly low nursing rate compared to the other strains and the offspring weaning rate also lower than both GF BABL/C and GF NSG. We hypothesize that the poor performance of KM mice as GF foster mothers might be attributed to two factors: their inherently higher body weight^20^ and the common occurrence of cecum enlargement following germ-free (GF) conversion^21^. When mothers squat on their pups in a nursing position, they provide them with belly heat and give them access to their nipples for sucking. During nursing, foster mothers squat over their pups, providing warmth and access to their nipples for feeding. However, in GF KM mice, increased body weight and an enlarged cecum may exert excessive physical pressure on the pups, leading to higher mortality and ultimately limiting their effectiveness as foster mothers.

Among the three inbred GF strains, all foster mothers provided adequate maternal care to cross-fostered pups within the first five days post-C-section. However, only half of the pups survived to weaning under GF C57 foster mothers, a stark contrast to studies on maternal care in SPF C57 foster mothers. SPF C57 foster mothers exhibiting more arched-back nursing, increased pup licking, and superior nest-building abilities compared to BC^11^. This discrepancy between SPF BC and C57 strains may be attributed to genetic differences, particularly allelic variations influencing anxiety-related behaviors. C57 mice are generally characterized as less emotionally reactive, whereas BC mice display heightened emotional reactivity^22^. Moreover, C57 mice raised by BC foster mothers display increased anxiety-like behaviors compared to those raised by their biological mothers, which correlates with elevated hypothalamic-pituitary-adrenal (HPA) axis activity^23^. These findings suggest that both genetic and epigenetic factors influence maternal behavior and contribute to behavioral differences in offspring. The gut-brain axis is increasingly recognized as playing a role in the pathophysiology of several neurological disorders, such as mood disorders, Parkinson’s disease, and others^24^. We hypothesize that microbiome depletion, particularly of gut microbiota, may influence anxiety-related behaviors in GF BALB/c foster mothers, potentially enhancing their maternal performance and contributing to improved weaning rates. Further research is required to validate this hypothesis. Finally, while NSG mice exhibited comparable maternal care to BALB/c foster mothers, the impact of their severe immunodeficiency on maternal behavior remains unclear. Future studies are required to determine whether immune system deficits influence maternal care and neonatal outcomes in NSG mice.

Our findings offer valuable insights into optimizing surgical techniques, donor selection, and fostering strategies to enhance neonatal survival and fostering success in GF mouse models. These improvements increased the efficiency and reproducibility of sterile cesarean procedures while minimizing batch variability and reducing mouse loss. However, the maternal environment significantly influences offspring behavior. Since most GF mice are obtained through cross-fostering, early maternal conditions may shape adult behavior and impact experimental outcomes. Therefore, careful consideration of maternal environment is essential in GF mouse breeding to ensure the reliability and consistency of research findings.

## Electronic supplementary material

Below is the link to the electronic supplementary material.

Supplementary Material 1

## Data Availability

All data is available within the manuscript.
