# Supplementary material for: Optimized cesarean techniques, IVF use, and foster strain selection enhance germ-free mouse production efficiency
Source: Sci Rep. 2025 Jul 26;15:27196. doi: 10.1038/s41598-025-05411-4 (PMC12297627; doi:10.1038/s41598-025-05411-4)
Supplement: Supplementary file 1 — Supplementary Material 1 [file 41598_2025_5411_MOESM1_ESM.docx]

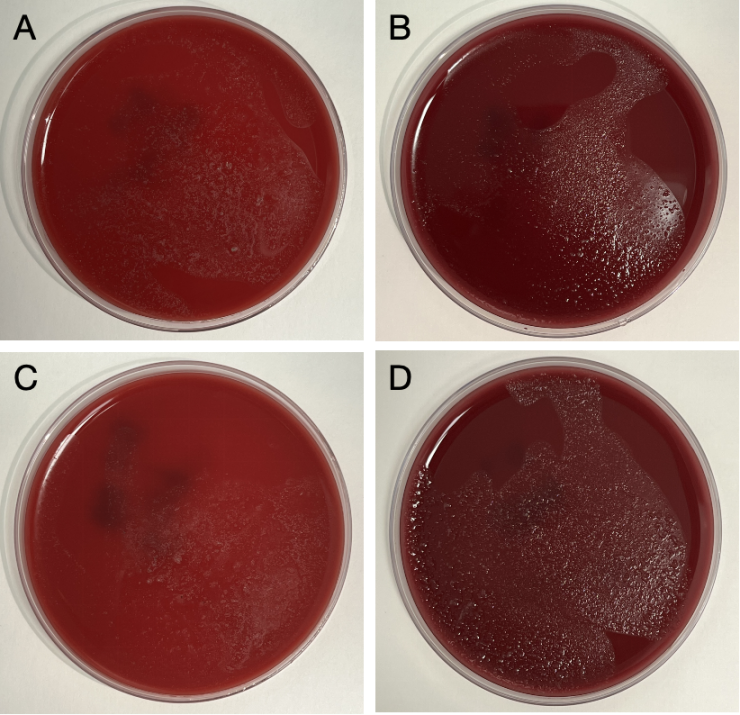


Figure 1: The culturing of fecal sample from germ-free mice after FRT-CS and T-CS. (A) Fecal of GF weanling pups obtained from FRT-CS was cultured in a Columbia blood agar plate under aerobic conditions for 48h. (B) Fecal of GF weanling pups obtained from FRT-CS was cultured in a Columbia blood agar plate under anaerobic conditions for 48h. (C) Fecal of GF weanling pups obtained from T-CS was cultured in a Columbia blood agar plate under aerobic conditions for 48h. (D) Fecal of GF weanling pups obtained from T-CS was cultured in a Columbia blood agar plate under anaerobic conditions for 48h.

| Mode of fertilization | Number of female mice | Form of copulatory plug  (G0.5) | | | | EDD | ADD | | | |
| --- | --- | --- | --- | --- | --- | --- | --- | --- | --- | --- |
|  |  | 0-24h | 24-48h | 48-72h | NA |  | G18 | G19 | G19.5 | NA |
| NM | 30 | 5 | 7 | 11 | 7 | G19.5 | 5 | 6 | 12 | 7 |
| IVF | 30 | / | | | | E19.5 | 0 | 2 | 28 | 0 |

Table 1: Predication of actual delivery date (ADD) for different fertilization models. NA for not available.


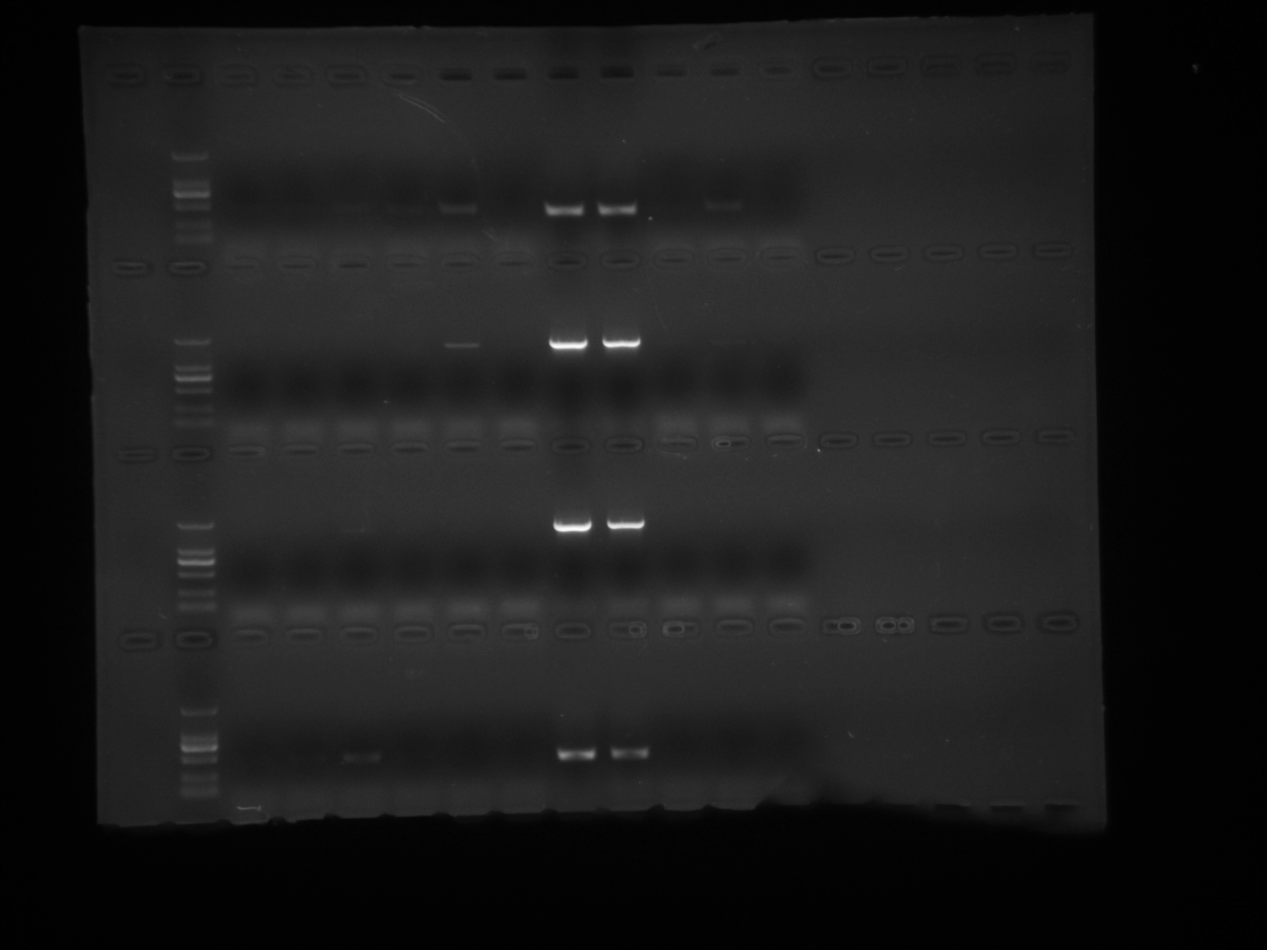


Figure 2：The complete membrane image depicting the detection of bacterial contamination following T-CS is presented in Figure 4 of the manuscript.


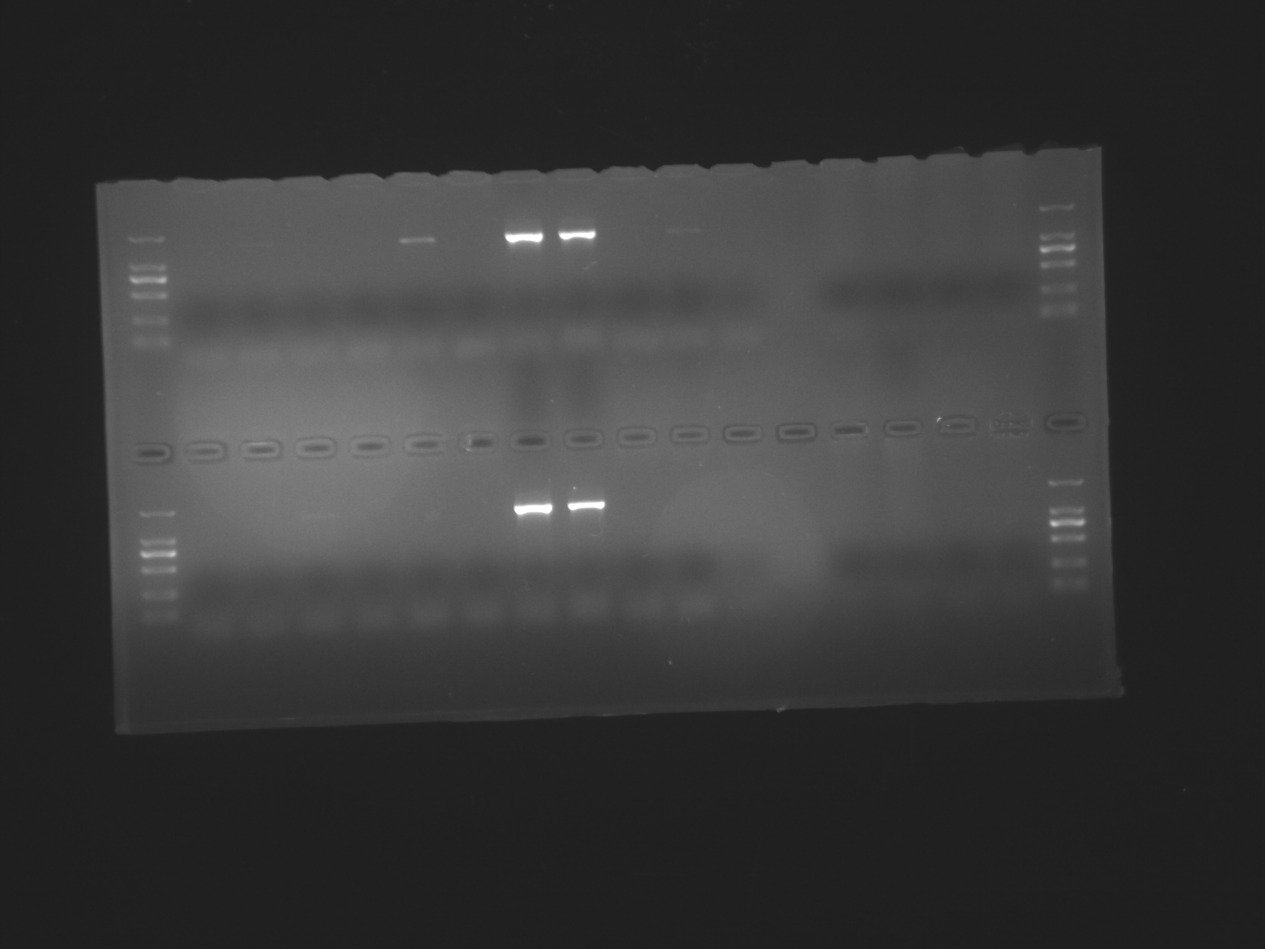


Figure 3：The complete membrane image depicting the detection of bacterial contamination following FRT-CS is presented in Figure 5 of the manuscript.

| Pathogenic Bacteria | |
| --- | --- |
| *Salmonella spp.* | *Pseudomonas aeruginosa* |
| *Mycoplasma spp.* | *Streptobacillus moniliformis* |
| *Corynebacterium kutscheri* | *Staphylococcus aureus* |
| *Tyzzer's organism* | *Streptococcus pnemoniae* |
| *Pasteurella pneumotropica* | *β-hemolyticstre ptococcus* |
| *Klebsiella pneumoniae* | *Citrobacter rodentium* |
| *Pneumocystis spp.* |  |
| Virus | |
| Hantavirus (HV) | Pneumonia Virus of Mice (PVM) |
| Mouse Hepatitis Virus (MHV) | Reovirus type 3 (Reo-3) |
| Sendai Virus (SV) | Minute Virus of Mice (MVM) |
| Ectromelia Virus (Ect.) | Polyoma Virus (POLY) |
| Lymphocytic Choriomeningitis Virus (LCMV) | Theiler’s Mouse Encephalomyelitis Virus (TMEV) |
| Parasites | |
| Ectoparasites | Ciliates |
| *Toxoplasma gondii* | All Helminths |
| Flagellates |  |

Table 2: The list of pathogenic bacteria, viruses, and parasites that SPF mice are required to be free from.
